# Knowledge, Attitudes, and Beliefs Regarding Medical Cannabis Among Patients and Providers in Florida’s Long-Term Care Facilities

**DOI:** 10.7759/cureus.66115

**Published:** 2024-08-04

**Authors:** Jennifer Attonito, Katherine Freeman, Melanie K Bone, Heather Howard, Carly Blum, George Luck

**Affiliations:** 1 Healthcare Administration, Florida Atlantic University Charles E. Schmidt College of Medicine, Boca Raton, USA; 2 Biostatistics Collaborative Core, Florida Atlantic University Charles E. Schmidt College of Medicine, Boca Raton, USA; 3 Obstetrics and Gynecology, Florida Atlantic University Charles E. Schmidt College of Medicine, Boca Raton, USA; 4 Sandler School of Social Work, Florida Atlantic University, Boca Raton, USA; 5 Political Sciences, University of Florida, Gainesville, USA; 6 Integrated Medical Science, Florida Atlantic University Charles E. Schmidt College of Medicine, Boca Raton, USA

**Keywords:** medical cannabis, long-term care, geriatric, medical marijuana, cannabis

## Abstract

Objectives: Medical cannabis (MC) has been found effective in treating multiple symptoms commonly experienced by older adults; however, residents in long-term care (LTC) often lack access to MC products. This study seeks to identify patterns and barriers to recommending MC to patients and to explore the knowledge and attitudes toward MC use among patients and providers.

Methods: The quantitative portion of this study employed a survey to assess the knowledge of, attitudes toward, and barriers to MC among 126 providers in Florida LTC. Frequencies were reported, bivariate associations were analyzed, and a final regression model predicting MC knowledge was tested. In-depth interviews were conducted with 25 LTC patients, and content was analyzed using the RADaR method.

Results: The age of the providers ranged from 21 to 74; 74% were female, 18% were Black/African American, and 17% reported Hispanic ethnicity. Less than half (37.2%) felt they received adequate training on MC. Having accurate knowledge about MC was associated with greater confidence in answering patients’ questions (p=0.002). Although most providers (94.2%) felt MC is a viable treatment option, the main barriers to recommending it to patients were a lack of proper training or clinical guidelines. Regarding patients, 16% reported ever using MC, and less than half (32%) had knowledge of MC or how to obtain products. Many believed it could help with symptoms and would consider its use if recommended by a doctor. However, they reported that MC was rarely recommended by providers and that they knew little about the use of this therapy.

Conclusion: This study underscores access challenges among seniors in LTC who might benefit most from MC’s therapeutic properties. Complex MC policy implementation issues are discussed. State and federal policy issues around cannabis contribute to limited research on the therapeutic uses of cannabis, as well as the MC access problem addressed in this study.

## Introduction

The medicinal properties of cannabis have been the subject of research and controversy for decades. Medical cannabis (MC) has been found effective in large-scale studies of patients to improve sleep, fatigue, anxiety, pain, anorexia, depression, muscle loss, labored breathing, nausea, and quality of life with few side effects [1,2]. Tetrahydrocannabinol (THC)-containing medicines are currently considered a third-line therapy in neuropathic pain by the European Pain Federation [3]. Some studies suggest that the cannabidiol (CBD) component of cannabis might help treat some psychiatric disorders, such as post-traumatic stress disorder, as it exerts anti-psychotic, anxiolytic, anti-inflammatory, and neuroprotective properties [1,4].

Older adults often suffer from chronic and complex conditions that can increase in intensity over time. In many cases, symptoms can be challenging to control, and opioids and other medications have adverse effects. Long-term care (LTC) is an essential principle in geriatric medicine. Its components focus on simplifying medication regimens, avoiding polypharmacy, and eliminating combinations of or single medications with potentially harmful side effect profiles [5]. LTC providers also aim to reduce the severity of anxiety and depression frequently experienced among older adults [6].

The Florida Agency for Healthcare Administration lists over 84,000 licensed nursing home beds and 106,103 assisted living facility beds. LTC residents frequently present with conditions and symptoms (e.g., chronic pain and insomnia) that MC could potentially manage [7]. However, limited evidence supports the health benefits claimed for this population. In clinical studies, half of the 10 most frequent chronic medical conditions coded for U.S. nursing home residents have symptoms that respond effectively to MC [8]. A 2020 study reported characteristics of 4,447 older adults in the Florida (FL) MC registry. The study found that upon FL’s MC program roll-out, more than half of early adopters were 50 years or older. The most common indicators were pain, musculoskeletal disorders, and spasms [9].

Federally, cannabis products have been designated as a Schedule I drug, which is considered to have no accepted medical use and a high potential for abuse. However, it is anticipated that the Justice Department will soon reclassify cannabis to Schedule III. Presently, cannabis is legal in 22 states for medical and recreational use, in 15 states for medical use only, and outlawed for any use in the remaining 13 states. Until the 2022 Medical Cannabis and Cannabidiol Research Expansion Act (H.R. 8454), clinical research on cannabis as a medical therapy had been limited by the substance’s illegal status. Laws concerning the cultivation, distribution, and procurement of cannabis vary widely by state in terms of implementation and enforcement. Where MC is legal, its cultivation, distribution, and access procedures remain inconsistent.

In FL, where MC is legal for qualified patients and recreational MC possession is a crime, interested adults must consult a state-certified physician and have a proven qualifying condition to obtain an MC card. MC products can then be procured through state-certified dispensaries. However, FL LTC residents face many logistical barriers to MC accessibility. According to an investigative news report, FL LTC residents admitted from home often have activities of daily living dependencies and moderate cognitive impairment, and many are not responsible for their own medical decisions [10]. For most nursing home patients, decision-making is left to guardians who may not be equipped or close enough to procure MC on the patient’s behalf. Per FL statute, only patients or their registered caregivers may handle the patient’s MC (note: caregivers must pass an exam and background check to qualify). Family members are not often available to take on this role, and LTC staff will not likely be listed as caregivers because most facilities have policies against staff using or accessing cannabis. Some dispensaries in FL will deliver to a residential facility, but the patient must meet the driver, show ID (which many LTC patients do not have), present their MC card, and pay for the product with cash. Many will only deliver for a minimum order of over $150, which is more product than would likely be needed for an elderly patient and more cash than many patients have [11]. Similar to LTC, an important study of acute care inpatient settings found that very few patients had access to their MC [12].

Both MC and nursing home oversight occur at the state level; however, a disconnect exists with federal policies that could improve MC access for states where it is legal. Cannabis's federally illegal status introduces regulatory concerns because healthcare facilities must comply with federal laws while balancing residents' rights to utilize MC. Research has revealed that clinicians are concerned about the conflicting state and federal laws on cannabis [13]. Although not common, some nursing homes have established MC policies to facilitate access. For example, Maine and Virginia passed bills ensuring nursing home patients' access to non-smoke MC when needed.

For LTC patients to benefit from the evidenced therapeutic advantages of MC, a better understanding of MC knowledge, attitudes, and utilization by providers and older adult patients in LTC institutionalized settings is needed. While there is some observational research pertaining to MC for seniors, less has been conducted for those residing in LTC facilities. Some investigators involved in this study work in the FL LTC environment and anecdotally report observing little to no recommendation of MC as patient therapy among FL geriatricians and nurse practitioners within LTC facilities. A 2018 study involving 426 palliative care providers concluded that despite their support for cannabis treatment of symptoms common in end-of-life care, less than half recommended cannabis to patients [14].

This analysis assessed degrees of knowledge of, attitudes toward, and barriers to the use of MC among providers and their older adult patients in FL LTC. An ecological perspective on health emphasizes both individual and contextual systems and the interdependent relations between the two. In the quantitative survey of providers, the primary variables of interest were knowledge about MC and the belief that MC is a viable medical therapy for some patients. Associations between these outcomes and other variables (confidence in recommending MC, attitudes toward MC, demographic characteristics, professional support, MC training, and barriers to recommending MC) were explored.

This study was designed to gain actionable insight into providers’ experiences in recommending MC for older adult residents in LTC and barriers to recommending MC. It also aimed to assess providers’ levels of knowledge and beliefs about MC. Further, the study team sought to identify factors associated with greater knowledge of MC and the belief that MC is a viable medical therapy. Qualitative interviews of residents in LTC were conducted to understand their knowledge and beliefs about MC and barriers to accessing MC. It is hoped that the findings provide evidence to guide MC access policies in this setting.

## Materials and methods

This study used a quantitative survey instrument among FL LTC healthcare providers. In addition, in-depth interviews were conducted with residents at FL assisted living facilities. The study protocol was approved by the university's institutional review board. All survey respondents and interviewees participated voluntarily and provided signed informed consent.

Quantitative survey: sample and data collection

Eligibility requirements included being 18 or older and working as a healthcare provider in a residential LTC in Florida. The provider sample in this study was accessed through the Society for Post-Acute & Long-Term Care Medicine in FL, the organization representing our provider population. The initial attempt to deliver the survey in person occurred at the November 2021 annual meeting of the Society. Less than a quarter of the needed surveys were acquired; due to COVID, turnout at this meeting was exceptionally low. The remainder of respondents were reached using an electronic (Qualtrics) version of the survey via email and indirectly through snowball sampling from December 2021 through March 2022, resulting in 126 surveys.

Qualitative interviews: sample and data collection

To identify LTC residents for interviews, researchers contacted assisted living facilities and nursing homes in South Florida. Many were hesitant to participate to protect their residents. Three FL LTC facilities (assisted living) allowed us to visit and invite residents to participate. Twenty-five participants were approached by study staff in common areas and asked if they were willing to participate in a brief (5-10 minute) interview. Those agreeing to participate were taken to a nearby private area to complete the interview.

Quantitative survey development

Survey questions pertaining to MC recommendation experiences, attitudes, and beliefs were adapted from existing instruments: one explored medical students’ knowledge, attitudes, and beliefs concerning MC use in a healthcare setting [15]; another measured oncologists’ experiences and attitudes around MC as an antiemetic for cancer patients [16]; and another examined family physicians' attitudes toward and experience recommending MC to patients [17]. Where question content was lacking in existing measurement instruments, de novo items were developed by methodologists and content experts in geriatrics and MC in FL. The survey was pilot-tested on a group of 10 LTC providers who were not included in the final study. Reviewer feedback was incorporated into the final version of the survey.

The survey included respondents’ demographics, professional roles, and sizes and types of LTC facilities. Questions asked about respondents’ perceived understanding of training around MC, interactions with patients about MC, beliefs about MC, knowledge about obtaining MC cards in FL, knowledge about MC treatment efficacy, and confidence in recommending MC to patients. A knowledge score was derived that assigned a ‘1’ for correct answers and a ‘0’ for incorrect answers. Points were added, accounting for the three items with reversed scoring. Other items addressed symptoms (e.g., pain, anxiety, nausea) and conditions (e.g., Parkinson’s disease, PTSD) where MC may be helpful, and the degree to which respondents felt MC could increase the risk of certain adverse events (e.g., falls, stroke, memory problems). Responses to knowledge questions were scored against current evidence of the clinical benefits of MC. “Correct” conditions were those listed as qualifying conditions to obtain an FL MC card. Questions pertaining to risks of MC use were also scored against current evidence, with “addiction” being the only evidenced risk (under medical supervision and not associated with other medications a patient might be using). The survey also asked respondents to select their greatest barriers to recommending MC to patients and their experience and future interest in recommending MC as a therapeutic agent for patients in LTC settings.

Qualitative interview development

A guide was developed for conducting semi-structured interviews with residents at FL assisted living facilities. Assisted living was chosen over nursing homes to improve validity by minimizing the risk of cognitive impairment. For inclusion, interviewees had to be residents in the LTC facility, displaying sufficient cognitive capacity to engage in a 5-10 minute conversation in English. Questions pertained to the use of MC, beliefs about MC effectiveness, attitudes toward MC, and interactions with physicians about MC as a therapy. Questions included: What do you know about the use of marijuana for medical concerns? Have you ever used marijuana for medical reasons? Would you consider the use of marijuana for health concerns? What symptoms do you feel could be taken care of with the use of medical marijuana? Have you ever approached a healthcare provider for use of medical marijuana? Has a medical provider ever suggested to you the use of marijuana for medical reasons? Would you consider the use of marijuana for health concerns if recommended by your healthcare provider? What do you think the process is for getting access to medical marijuana? For questions that yielded yes/no responses, interviewers probed for additional information. After twenty-five interviews, data saturation was reached. Interviews were recorded and evaluated by two investigators to validate the interpretation of responses. Qualitative rigor was evaluated using the COREQ (COnsolidated criteria for REporting Qualitative research) Checklist.

Data management and analysis

Categorical variables are presented as relative frequencies. Years of experience among providers were dichotomized at 10 years, and the age of residents was categorized at 65 years to reflect those entitled to Medicare benefits. Other continuous variables were dichotomized near their medians. For descriptive purposes, Likert scale items assessing self-perceived knowledge about MC and beliefs about MC were dichotomized based on whether the respondent “strongly agreed” or “somewhat agreed.” Likert scale items assessing knowledge about MC effectiveness in treating symptoms were dichotomized to include responses of “extremely helpful” or “very helpful.” In bivariate and multivariate analyses, continuous scales for these variables were used as such.

Data missingness varied by variable and case. Little’s test of missingness was conducted, and it was ascertained that the data were MCAR (p = 1.00). The association between categories of certification and knowledge score was tested using an analysis of variance with a Tukey multiple comparison procedure. Analyses were repeated after incorporating multiple imputation methods for missing data for knowledge scores, with checks for missingness. Associations between knowledge scores and preparedness, attitudes, beliefs, categorical demographic characteristics, practice behaviors, and barriers to recommending MC were tested using Wilcoxon rank sum or Kruskal-Wallis tests. Associations between knowledge scores and ordinal variables concerning attitudes and beliefs were analyzed using Spearman rank correlations. For bivariate associations between knowledge scores and other variables, variables yielding p-values less than 0.20 were incorporated into an initial multiple regression model. The model was run, and the variable with the highest non-significant p-value was eliminated; this procedure was repeated until only significant (p<.05) variables remained. All analyses were two-tailed and performed using SAS (Version 9.4, SAS Institute, Cary, North Carolina).

Qualitative data were analyzed using the comprehensive qualitative analysis strategy called the rigorous and accelerated data reduction (RADaR) technique used for applied research. The RADaR technique converts raw, textual data into a more manageable format. Systematic analysis occurring during each step of the process ensures rigor.

## Results

Survey

Demographic Overview

Descriptive statistics are shown in Table 1. Of the survey respondents (N=126), the largest group were RNs or nurse practitioners. Other categories of providers included pharmacists, physicians, and administrators, such as medical directors and nursing directors. Ages ranged from 21 to 74 years, with 75% being 30 to 65 years old, 74% were female, 18% were Black/African American, and 17% reported Hispanic ethnicity. The size of the facilities where respondents worked was split evenly between <150 beds and 150+ beds.

**Table 1:** Sociodemographic characteristics of clinicians and administrators (N= 126) in Florida long-term care facilities. Note that categories may not total the full sample size (126) due to missing data.

Sociodemographic Characteristics	N	%
Clinical title		
Nurse (RN)	21	24.42
Nurse practitioner	11	12.79
Other clinician	36	41.86
Pharmacist	1	1.16
Physician	17	19.77
Administrative title		
Nursing director	6	6.38
Medical director	11	11.70
Not applicable	45	47.87
Other administrator	32	34.04
Age		
Under 30 years	12	14.12
30 to 65 years	64	75.29
65 and older	9	10.59
Gender		
Female	71	73.96
Male	25	26.04
Nonbinary or other	0	0.00
Race		
Asian	2	2.27
Black/African American	16	18.18
White/Caucasian	62	70.45
Do not wish to answer	3	3.41
Other race	5	5.68
Hispanic ethnicity		
No	70	83.33
Yes	14	16.67
Facility size: number of beds		
<150 beds	32	50.00
150+ beds	32	50.00
Facility size: number of residents		
<150 residents	25	54.35
>150 residents	21	45.65

Self-Perceived Knowledge and Beliefs

Less than half of the providers felt they received adequate training on MC (37.2%), had experience discussing the indications and contraindications of MC with patients and/or caregivers (28.6%), and were confident they could answer patients’ and/or caregivers’ questions about MC (34.5%). Most (68.2%) stated they understood the difference between THC and CBD, and 70.1% were aware of the different routes of administration. Nearly all felt that MC is a viable treatment option (94.2%) and that MC should be federally legal (81.2%). They also reported concerns: patients might self-treat with street cannabis (71.4%), MC can interact with other medications (54.8%), and use can lead to addiction (22.6%). About a third agreed that there is not enough MC research (36.5%) (Table 2).

**Table 2:** Self-perceived knowledge and beliefs about MC among Florida long-term care providers (N=126) MC: medical cannabis, THC: tetrahydrocannabinol, CBD: cannabidiol.

Statement	N	% Strongly or Somewhat Agree
Self-perceived knowledge		
I have received adequate training on medical cannabis.	86	37.21
I understand the difference between THC and CBD components of medical cannabis products.	85	68.23
I am aware of the different routes of administration for medical cannabis.	77	70.13
I have experience in discussing the indications and contraindications of medical cannabis with patients and/or their caregivers.	84	28.58
I am confident I can answer patients’ and/or caregivers’ questions about medical cannabis.	84	34.53
I know how to obtain a medical cannabis card in the state of Florida.	84	47.62
Beliefs		
MC is safe	87	70.12
MC is a viable treatment option	86	94.19
All clinicians should be trained	85	94.12
MC should be recommended to patients	86	77.91
MC should be federally legal	85	81.18
Patients might self-treat with street cannabis	84	71.43
MC can interact with other meds	84	54.76
Not enough MC research	85	36.47
Use of MC can lead to addiction	84	22.62
The MC certification process is difficult for patients	84	33.33

Knowledge of MC for Symptoms and Conditions

Most respondents accurately identified symptoms known to benefit from MC therapy: anxiety (70.1%), appetite loss (80.8%), chronic pain (72.3%), insomnia (61.4%), and nausea (67.1%). However, some respondents inaccurately selected symptoms that are not known to respond to MC, including acute pain (66.7%), cold/flu (15.1%), and depression (52.1%). Very few selected conditions not known to respond well to MC therapy: chronic obstructive pulmonary disease (7.1%), diabetes (4.0%), heart failure (2.4%), and upper respiratory infections (1.6%) (Table 3).

**Table 3:** Knowledge about MC effectiveness in treating symptoms and medical conditions among FL long-term care providers (N=126) *Evidence indicating that MC has no effect on these conditions. MC: medical cannabis, ALS: amyotrophic lateral sclerosis, COPD: chronic obstructive pulmonary disease, IBD: inflammatory bowel disease, MS: multiple sclerosis, PTSD: post-traumatic stress disorder.

Symptoms and Conditions	N	% Who Answered Extremely/Very Helpful
Knowledge: MC effectiveness in treating symptoms		
Acute pain	75	66.67
Anxiety	77	70.13
Appetite loss	73	80.83
Chronic pain	50	72.37
Cold/flu	53	15.10
Depression	71	52.11
Insomnia	70	61.43
Nausea	64	67.19
Knowledge: MC effectiveness in treating conditions		
ALS	29	23.02
Alzheimer's disease	20	15.87
Cancer	54	42.86
COPD*	9	7.14
Depression	47	37.30
Diabetes	5	3.97
Epilepsy	34	26.98
Glaucoma	37	29.37
Heart failure*	3	2.38
HIV/AIDS	16	12.70
IBD	27	21.43
MS	31	24.60
Parkinson's disease	37	29.37
PTSD	53	42.06
Seizures	40	31.75
Upper respiratory infections*	2	1.59
Other serious terminal conditions	39	30.95

Risks of MC Treatment

The most frequently selected risks were falls (27.8%), memory problems (23.0%), psychosis (15.9%), addiction (12.7%), and drug overdose (10.3%), with very low prevalence (<10%) for the remainder of the listed risks (Table 4).

**Table 4:** Knowledge about risks of using MC among Florida long-term care providers (N= 126) MC: medical cannabis.

Knowledge: MC Risks	n (N = 126)	% Who Answered Yes
Addiction	16	12.70
Cancer	3	2.38
Depression	18	14.29
Diabetes	1	0.79
Drug overdose	13	10.32
Falls	35	27.78
Heart attack	4	3.17
Memory problems	29	23.02
Psychosis	20	15.87
Respiratory symptoms	13	10.32
Nausea/vomiting	10	7.94
Stroke	4	3.17

Barriers to MC Recommendation

The top barriers to MC recommendation were not having enough training to help patients (30.9%), lack of clinical guidelines (38.6%), lack of patient education tools (20.6%), lack of support from medical assoc./licensing bodies (14.3%), risk of losing reimbursement from Medicare/Medicaid (14.3%), and lack of support from other direct care staff (10.3%) (Table 5). 

**Table 5:** Barriers to recommending MC for patients among providers in Florida long-term care facilities (N= 126) MC: medical cannabis.

Barriers	n (N = 126)	% Who Answered Yes
Not enough training	39	30.95
Lack of patient educational tools	26	20.63
Lack of clinical guidelines	36	28.57
Lack of support from medical assoc./Licensing bodies	18	14.29
Availability of Rx cannabinoids	12	9.52
Poor or diminished patient comprehension of Florida certification process	6	4.76
Lack of patient interest	2	1.59
Patient self-dosing	12	9.52
Concern patients may use recreationally	6	4.76
Patient challenges to MC certification	8	6.35
Patient challenges to accessing MC products	10	7.94
Potential liability concerns	12	9.52
Risk of losing reimbursement from Medicare/Medicaid	18	14.29
Time constraints	1	0.79
Lack of support from other direct care staff	13	10.32
Facility location or other structural limitations	7	5.56
Other	10	7.94

MC Certification Status as Providers

Among physicians surveyed, 4.1% reported being certified to recommend MC to patients and 24.5% of those not certified reported interest in becoming MC certified in FL. Although the majority (69.49%) reported awareness that some patients use MC and that 78.95%) reported patients have requested it, only about half (50.98% of providers have suggested MC to patients.

Quantitative: bivariate and multivariate analysis

Factors Associated With MC Knowledge

In the multivariate final model, higher knowledge scores were significantly associated with higher levels of confidence in answering patients’ and/or caregivers’ questions about MC (regression coefficient = -0.93815, SE = 0.23884, p = 0.0002; bivariate Spearman coefficient = -0.33146, p = 0.0021). Additional variables significantly associated with higher knowledge scores were respondents hesitating to recommend MC because they did not have enough training to help patients (regression coefficient = 1.41043, SE = 0.70550, p = 0.0490; bivariate Wilcoxon Rank Sum Z = 3.8762, p = 0.0001) and because of a lack of support from medical associations and licensing bodies (regression coefficient = 3.15122, SE = 0.78605, p = 0.0001; bivariate Wilcoxon Rank Sum Z = 5.1753, p < 0.0001).

Factors Associated With the Belief That MC Is a Viable Therapy

From Spearman rank correlations, the belief that MC is a viable therapy was significantly and positively correlated (p< 0.05) with degree of agreement in having received adequate training on MC (r=0.26), understanding differences between MC and CBD (r=0.33), awareness of different routes of MC administration (r=0.39), confidence in answering patients’ or caregivers’ questions about MC (r=0.29), and knowing how to obtain an MC card in FL (r=0.40).

Differences in Beliefs by Sex and Ethnicity

Secondary analyses found that females were more likely to indicate that MC is helpful in treating acute and chronic pain (p=0.04, p=0.049, respectively). Females were more likely to have reported that healthcare professionals should receive training in the use of MC (p<0.01) and were more likely to agree with the premise that MC can lead to addiction (p=0.04). Hispanic respondents were more likely to disagree that providers should recommend MC for some conditions (p=0.04) and that MC could have concerning interactions with other therapies (p=0.0402).

Qualitative analysis

Patients (N=25) included White non-Hispanic females (n=17) ranging in age from 70 to 99 years, and White non-Hispanic males (n=8) ranging in age from 85 to 93 years. Only 32% had knowledge of the use of MC; 16% had used MC; 48% believed it could help with Parkinson's, chronic pain, uneasiness, depression, uncomfortable symptoms, panic attacks, and arthritis; 68% would consider use if recommended by a provider; and 28% had some knowledge of how to obtain MC products. Some participants considered MC and recreational cannabis use to be similar. The primary barriers reported were that it is not recommended by providers, there is a lack of knowledge about MC, and not knowing how to access MC products. The following are examples of participants’ quotes to demonstrate the three major themes.

**Table 6:** Participants’ quotes demonstrating three major themes

Themes	Quotes
Variability of MMJ usage	[MMJ] would calm down my anxiety. I have severe panic attacks… I’ve been here over a month, and I still can’t stand or walk. My shoulders and the knees and the back are shot, so I’m going home Friday and I won’t be able to stand or walk. So I figured it might give me maybe some confidence or feel a little strength in my body to help me at home.
	I am sure that depression which is prevalent amongst a lot of people here can be helped. I’m sure that someone like me with peripheral neuropathy - it can be helped by also. And my daughter who is a physician doesn’t want me having medical marijuana.
	If it helps with pain, I’ll use it.
Process of obtaining MMJ	Expensive but a simple process. My doctor was lovely.
	I hear it is not easy, lots of red tape. Need a doctor’s OK and more processes.
	In Florida you’re required to take out a permit, which I still have, to be able to buy it. You can’t buy it any other way. You have to go through the state, pay them $77, and they send you a card.
Hopeful for the use of MMJ	I have heard good things about it.
	I would do it if necessary and in pain.
	It can be beneficial if used properly.
	I do know that it could be good for certain local diseases and conditions and that others work better in other areas.

## Discussion

This study aimed to assess the knowledge, beliefs, and barriers faced by Florida LTC providers regarding the recommendation of MC to patients. It also sought to understand the sociodemographic factors influencing providers' attitudes toward MC and to identify potential gaps in training and clinical guidelines.

FL LTC providers in this study reported not recommending MC to patients mainly due to a lack of clinical guidance. While about a third of providers felt confident in talking with patients and caregivers about MC as a possible therapy for those who might benefit, issues of medical training were the top-listed barriers to recommending MC for patients. Providers in this study were accurate in their assessments of many conditions and symptoms known to respond to MC; however, they occasionally selected inaccurate items, such as treatment for acute pain and depression. Surprisingly few providers (12.7%) identified addiction as a risk, given that important research findings point to cannabis use disorder as a problem for many MC users [18]. It was not surprising that higher MC knowledge scores were correlated with higher confidence in recommending MC to patients and that nearly 100% of respondents felt all clinicians should receive training on MC. The LTC clinicians who believed MC to be a viable treatment option also expressed greater confidence in recommending MC to patients, a greater understanding of accessing MC products, and a higher likelihood of having received MC-related training.

Regarding demographics, female LTC providers were stronger proponents of MC for pain relief. Results exploring gender differences in attitudes toward MC have been mixed. Research has shown that women are more likely to report pain, possibly explaining the gender difference observed here [19]. Hispanic providers in this study were stronger opponents of MC overall. This finding was surprising, as a recent study found MC use to increase among Hispanics in states where MC was legal [20]. However, research has shown that substantial stigma against drug use, including cannabis, exists among Hispanic American populations [21].

This study found that MC-related experience and confidence were associated with accurate knowledge and positive beliefs about MC. Overall, the majority of clinicians reported having suggested MC to patients, and 22% expressed interest in becoming MC certified to recommend MC in the State of Florida. Furthermore, many felt that MC should be federally legalized for medical purposes and that providers should recommend MC where appropriate. Hence, positive attitudes toward MC as a viable therapy for some patients and an interest in learning more about MC appeared to be important findings in this study.

In qualitative interviews, patients in this study also had positive attitudes toward MC, but few were clear about how to access MC products. The reported need for patient education tools on MC was echoed among the patients in this study. Though few patients said they had tried it, most stated that they would consider using MC if their physician recommended it. However, it seems that MC as a treatment option is not often addressed in consultations. As quoted by an interviewee who was hesitant to consider the use of MC, “my daughter who is a physician doesn’t want me having medical cannabis.” However, as noted by an older adult participant, MC may be an option to suggest when screening for mental health symptoms such as anxiety and depression.

This study found that major barriers to recommending MC for patients were lack of training, clinical guidelines, and patient education tools. These findings align with previous research. According to a systematic review published in JAMA, cannabis use among older adults in the U.S. has steadily increased [22]; however, most providers in this study expressed a lack of interest in seeking state certification to become MC practitioners. Studies show that medical students do not receive formal training related to recommending MC or managing its use [23]. Physicians who recommend MC to patients report that clinical factors are important in making MC recommendations; however, assessment practices and treatment regimens vary greatly and rely on non-evidence-based approaches [24].

Among gerontologists and providers treating older adults, additional challenges exist. A survey of gerontology residents found that most indicated they had no formal education about MC and reported being unprepared to answer clients’ MC-related questions [25]. Similarly, palliative care providers report positive beliefs around MC but remain reluctant to recommend cannabis products to patients [14]. The majority of MC utilization research has been conducted among oncologists and cancer patients. In a national survey of 400 oncologists’ attitudes toward and knowledge of MC, most reported discussing MC with patients or their families; however, only 30% reported feeling adequately knowledgeable to recommend MC, and more than half of those who made recommendations considered themselves ill-equipped [24]. Among both providers and patients, there is a distinct lack of in-depth understanding of the protective and risk properties of cannabis, especially for older adults. For example, pain is the most cited “medical” reason for MC use [26]. However, the International Association for the Study of Pain does not endorse general cannabis use for pain relief due to limited evidence [27].

Policy implications

Cannabis remains illegal at the federal level; however, the Justice Department is moving forward on reclassifying the substance to Schedule III, indicating advancing acceptance of cannabis as a drug with a lower potential for abuse. Most states have decriminalized MC use for specific diagnoses, many of which are prevalent among assisted living and nursing home residents; however, regulatory concerns remain as many skilled nursing facilities and other LTC organizations must comply with federal laws, particularly regarding Medicare. These structural limitations do not incentivize the LTC community to seek credentialing or make efforts toward policy change. In addition, research has also found that, from the physician's perspective, there is a stigma against becoming a certified MC clinician [28]. According to an investigative report in FL, physicians may fear being blacklisted by hospital systems, nursing homes, or hospices, which rely on Medicaid and Medicare reimbursement funds [29]. Further, although cannabis is only legal for medical purposes in FL, MC dispensaries espouse more euphoric than therapeutic effects of their products, likely contributing to the stigma surrounding this substance as a legitimate medical treatment. The disconnect between the state’s required “medical” recommendation by a physician, the storefront sales by non-medical sellers, and the marketing of cannabis’ “high” likely explain some of the stigma expressed by this study’s participants in the LTC medical community. Regardless of such resistance, representatives of The Society for Post-Acute and Long-Term Care Medicine advocate for the inclusion of cannabis products as a complementary therapy for LTC residents [5]. If physicians are to feel confident and effective in recommending MC to patients, barriers to initiating MC research must be addressed. A 2021 monograph described three chief barriers to initiating research geared toward addressing this highly pressing public health issue: the US regulatory status of cannabis and cannabinoids, sources for cannabis and cannabinoid study medications, and limited funding and resources to support studies [30].

Limitations

This study had several clear strengths, including the diverse provider demographic and the mixed-methods approach; however, it is not without limitations. The sample size was 30% of the target. Data were collected within FL, with policies specific to this state, and only in a subset of LTC settings. Outcomes may only generalize within the state or to states with similar MC legislation. It is recommended that future studies be conducted with a larger sample in other regions of the U.S. The quantitative survey was open to doctors, nurses, therapists, and administrators; therefore, it may be difficult to extrapolate whether results can promote change in clinical care. The Hispanic population of providers was treated as one group for analysis, even though there are cultural differences according to country of origin. Furthermore, self-report bias must be considered when conducting survey research about controversial or stigmatized subject matter. It should also be noted that in the qualitative portion of this study, respondents were highly homogeneous (all White, non-Hispanic), and self-selection bias may exist for interviews conducted. It is recommended that future qualitative studies on this subject include a more diverse sample of respondents. Finally, readers should keep in mind that state and federal cannabis legislation is evolving rapidly, and the information reported here may change.

## Conclusions

Results from this study and other research highlight significant disconnects between current MC policy at state and federal levels, patient access to MC products, and available guidelines and training for physicians. As evidence emerges, supportive clinical outcomes for MC as a therapeutic agent must be communicated to trained physicians and medical students.

As found in this study, many clinicians feel uninformed and unprepared to include MC in treatment regimens for older adult patients. Rather than subjecting MC to the same drug development rigor applied to allopathic medications, a paradigm shift is necessary in how we manage alternative and complementary medications. Finally, a team approach, including the patient, an MC management practitioner, medical providers, and hands-on caregivers, can support access to and implementation of high-quality and thoughtful MC care, resulting in the best outcomes for older patients.
